# Bortezomib-induced Severe Congestive Heart Failure

**DOI:** 10.4021/cr105e

**Published:** 2010-11-20

**Authors:** James H. Jerkins, Anca Suciu, Sula Mazimba, Alejandro Calvo

**Affiliations:** a Department of Graduate Medical Education, Kettering Medical Center, USA; b Medical Oncology and Hematology, Kettering Medical Center, USA

**Keywords:** Myeloma, Congestive heart failure, Bortezomib

## Abstract

The clinical manifestations of anti-cancer drug associated cardiac side effects are diverse and can range from acutely induced cardiac arrhythmias to severe contractile dysfunction, and potentially fatal heart failure. Anthracyclines and trastuzumab cardiac toxicity have been well described and left ventricular ejection fraction (LVEF) evaluation is commonly performed before their use. Bortezomib (Velcade), a potent, specific and reversible proteasome inhibitor is approved for treatment of multiple myeloma (MM). The incidence of cardiac failure associated with bortezomib therapy in clinical trials remains incidental. Acute exacerbation of pre-existing congestive cardiac failure has been associated with this therapy but *de novo* cardiomyopathy has been reported in only one patient receiving bortezomib for small cell lung cancer. As a result, cardiac evaluation is not normally ordered before its use. We describe a 50-year-old female with newly diagnosed MM and no risk factors for cardiac disease that unexpectedly developed florid heart failure after 2 cycles of bortezomib and low-dose dexamethasone. 2-D echocardiogram showed dilated cardiomyopathy with severely decreased LVEF; no changes consistent with amyloid deposits or myocardial scarring were described. Coronary angiogram ruled out coronary artery disease. The mechanism of bortezomib-induced cardiomyopathy has been postulated to be through fluid retention. Based on literature review we hypothesize that the disruption of the ubiquitin-proteasome system by bortezomib may cause cardiomyopathy and severe cardiac failure. As Bortezomib is a new and promising therapy for MM patients, we recommend routinely monitoring cardiac parameters in patients undergoing this treatment.

## Introduction

Multiple Myeloma, a neoplastic proliferation of an aberrant colony of plasma cells, has historically been difficult to treat with a previous complete remission rate of less than five percent [1]. Subsequent research has identified the ubiquitin-proteasome pathway (UPP), represented by ubiquitin-conjugating system and the proteasome, as a possible target for chemotherapeutic agents secondary to the role it plays in the degradation of cellular proteins and basic processes of eukaryotic cells [2]. Inhibition of the proteasome, key constituent of UPP, consequently blocks cellular growth and division by multiple pathways, ultimately leading to a pro-apoptotic state [2].

Bortezomib, a dipeptidyl boronic acid, is a potent, specific and reversible inhibitor of the chymotrypsin-like activity of the 26S proteasome [3], approved for treatment of Multiple Myeloma [4-6]. The drug is also under investigation for the treatment of other hematologic cancers, such as specific types of lymphoma, and a variety of solid tumors, including prostate, lung, breast and ovarian cancer [7, 8]. Bortezomib functions by blocking the proteolytic degradation of IĸB, the inhibitor of nucleus factor ĸB (NF-ĸB) [6]. NF-ĸB acts as a transcription factor, turning on genes that cause production of proteins implicated in cell growth, surface molecules which allow myeloma cells to stick to cells in bone marrow and stimulate bone marrow to produce vascular endothelial growth factor, promoting angiogenesis. By inhibiting the breakdown of IĸB and subsequent inhibition of NF-ĸB, bortezomib leads to inhibition of cell growth and promotion of apoptosis in cancer cells [9].

In the SUMMIT and CREST phase II trials, the most common grade 3 and 4 adverse effects in MM patients receiving bortezomib included thrombocytopenia, fatigue, peripheral neuropathy and neutropenia [5, 6]. Interestingly, less than 5% of MM patients enrolled in these trials experienced grade three or four dyspnea or edema [5, 6]. An extension study based on the SUMMIT and CREST trials documented one case of cardiomegaly; however, this event was attributed to underlying or predisposing diseases [10]. The APEX trial, a phase III trial, reported seven patients who developed congestive heart failure; however, this was similar to rates of congestive heart failure in the dexamethasone arm [11]. Additionally, the patients in these trials had been previously treated with multiple cycles of chemotherapeutic agents including anthracyclines [5] which carry risks of cardiac toxicities [12]. In a phase III study using bortezomib as a first line agent combined with melphalan and prednisone, no incidence of cardiomyopathy was documented and the incidence of peripheral edema and dyspnea was 68% and 34% respectively, consistent with previously documented rates [4]. In another phase II trial in mantle cell lymphoma patients receiving bortezomib, Assouline and colleagues reported five cases of severe fluid retention in patients with baseline dyspnea or peripheral edema, making the authors amend their study to exclude patients with baseline fluid retention [13].

Only one case of bortezomib-induced cardiomyopathy with severely decreased left ventricular ejection fraction (LVEF) has been described in the literature in our knowledge. In their case report, Voortman and Giaccone described a patient with non-small cell lung cancer treated with four 3-week cycles of bortezomib 1.0 mg/m^2^ combined with cisplatin and gemcitabine who subsequently developed significant heart failure. In the conclusion, Voortman and Giaccone hypothesized that the presence of sub-clinical cardiomyopathy and inhibition of the UPP could predispose their patient to severe cardiac side effects [14].

## Case Report

A 50-year-old female patient diagnosed with stage IIIB Multiple Myeloma by Durie-Salmon criteria who was subsequently treated with bortezomib combined with low dose dexamethasone. The patient received two 3-week cycles of bortezomib 1.3 mg/m^2^ on days 1, 4, 8, 11 of each cycle and low dose dexamethasone.

The disease had a good response to therapy with normalization of serum electrophoresis. The patient developed tumor lysis syndrome and acute chronic renal failure after the first cycle of therapy with recovery of the renal function to previous values. During the entire course of the treatment, the patient had been on direct oncologic supervision and no new adverse effects were noticed.

However, after the second cycle of therapy the patient reported increased fatigue, severe dyspnea, orthopnea and mild lower extremity edema. She did not experience any chest pain or myalgia. The patient had no documented history of coronary artery disease, hypertension, dyslipidemia or pre-existing congestive heart failure. She had no history of smoking, alcohol or drug abuse.

The physical examination of the heart elicited a lateral displacement of apical impulse without any auscultatory abnormalities. Pulmonary auscultation revealed bi-lateral basilar crepitation. Central venous pressure as determined by neck vein distention was elevated at 10 cm and no hepato-jugular reflux was noted. There was mild peripheral pedal edema up to ankles bilateral.

**Figure 1:**
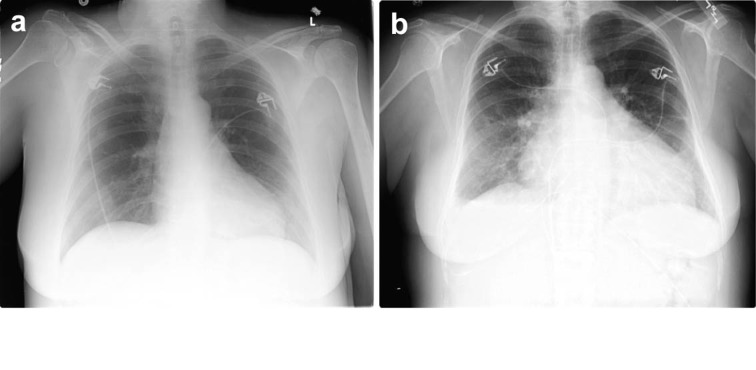
(a) Routine chest X-Ray at the diagnosis of MM; (b) Cardiomegaly 3 months after.

An ECG did not show signs of ischemia. The chest X-Ray showed an increased heart-chest ratio of 0.56, compared with 3 months old baseline of 0.47, and mild signs of pulmonary vasculature congestion (Fig. 1).

**Figure 2:**
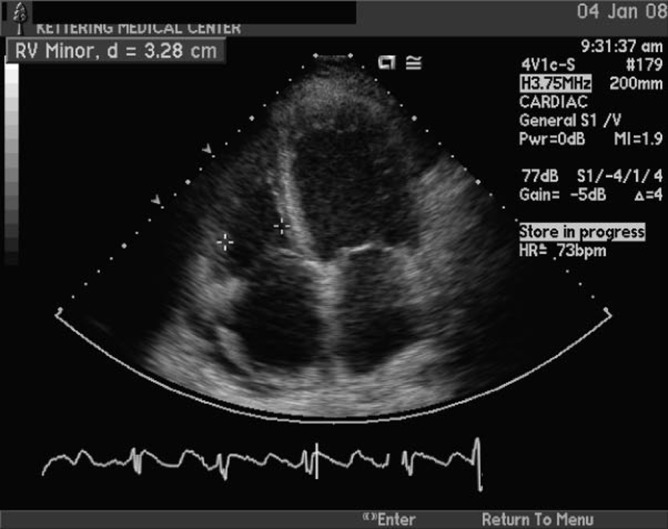
2D echo-cardiogram. Four-chamber view shows atrial and ventricular dilation.

Troponin as well as CK-MB levels were not elevated in the serial blood tests. The brain natriuretic peptide (BNP) was high at 979. She was diagnosed clinically with congestive heart failure by Framingham criteria and improved remarkably upon administration of furosemide. All chemotherapy was discontinued and being considered as a possible cause for fluid overload. The 2D echo-cardiogram performed on the day following admission showed severe decreased LVEF to less than 10-15%, mild bi-ventricular and bi-atrial enlargement, and severe global hypokinesis (Fig. 2). No changes consisting with prior ischemic event (myocardial scaring) or amyloid deposition were present.

The patient underwent heart catheterization which showed severe systolic dysfunction without evidence of coronary artery disease, and was diagnosed with New York Heart Association (NYHA) class III congestive heart failure. She was treated with furosemide, beta-blockers, hydralazine and digoxin, and discharged upon symptomatic improvement. A follow-up 2-D echocardiogram, one month after the initial diagnostic echo, showed an improvement of the systolic function with an improvement in her EF to 30%.

## Discussion

Research demonstrates that in long-term survivors of cancer, cardiovascular disease is one of the leading causes of morbidity and mortality. This is generally attributed to anti-cancer drugs’ wide range of cardiac side effects ranching from induction of cardiac arrhythmias to severe contractile dysfunction, and fatal heart failure [12]. The cardiac related toxicities of the anthracycline family of agents as well as Trastuzumab have been well described and LVEF evaluation is commonly performed before their use [12, 15]. However, when bortezomib was approved by the federal drug administration in 2003, no recommendations were made regarding cardiac monitoring [16].

The ubiquitin-proteasome system has been shown to play a significant role in the biology of eukaryotic cells through its degradation of cellular proteins [17]. Inhibition of the ubiquitin-proteasome system has been shown to lead to hyperubiquitination of intracellular proteins [18]. Subsequent cellular studies of dilated cardiomyopathy have shown accumulation of hyperubiquitinated proteins within the myocytes [19-21] secondary to proteasome inhibition [20]. Current literature has shown that, through the hyperubiquitination of key cell cycle regulatory proteins, the pro-apoptotic/anti-apoptotic ratio within the cell is altered leading to the development of cardiomyopathy [20].

We attribute the severe reduction of the LVEF in this case to the drug therapy with bortezomib. The negative findings of coronary angiography and the lack of cardiac risk factors exclude ischemia as the cause for congestive heart failure. The patient had no prior documented history or symptoms of heart disease and the new onset cardiomegaly on heart chest X-Ray, in only three months, rules out pre-existing dilative cardiomyopathy. The lack of fever, chest pain, normal EKG and cardiac enzymes, and the clinical course made the diagnosis of viral myocarditis unlikely. Relative newly diagnosed MM in the absence of typical myocardial changes like hypertrophy, granular sparking infiltrates on the 2D echocardiogram excludes amyloid deposition from the etiology of this ‘*de novo*’ dilative cardiomyopathy.

## Conclusions

We hypothesize that the cardiomyopathy in our patient was induced by Bortezomib through inhibition of the ubiquitin-proteasome system. The improvement of EF after just one month from the diagnosis and treatment of congestive heart failure may prove the reversibility of the effect of proteasome inhibition by this chemotherapeutic drug. As bortezomib is a new and promising therapy for multiple myeloma we recommend close monitoring of the cardiac parameters in patients undergoing this therapy.
